# Identification of *Wolbachia*-responsive microRNAs in the two-spotted spider mite, *Tetranychus urticae*

**DOI:** 10.1186/1471-2164-15-1122

**Published:** 2014-12-16

**Authors:** Xia Rong, Yan-Kai Zhang, Kai-Jun Zhang, Xiao-Yue Hong

**Affiliations:** Department of Entomology, College of Plant Protection, Nanjing Agricultural University, Nanjing, Jiangsu China; Department of Entomology, College of Plant Protection, Southwest University, Chongqing, Chongqing China

**Keywords:** Two-spotted spider mite, *Wolbachia*, miRNAs, Gene expression

## Abstract

**Background:**

The two-spotted spider mite, *Tetranychus urticae*, is infected with *Wolbachia*, which have the ability to manipulate host reproduction and fitness. MicroRNAs (miRNAs) are small non-coding RNAs that are involved in many biological processes such as development, reproduction and host-pathogen interactions. Although miRNA was observed to involve in *Wolbachia*-host interactions in the other insect systems, its roles have not been fully deciphered in the two-spotted spider mite.

**Results:**

Small RNA libraries of infected and uninfected *T. urticae* for both sexes (in total four libraries) were constructed. By integrating the mRNA data originated from the same samples, the target genes of the differentially expressed miRNAs were predicted. Then, GO and pathway analyses were performed for the target genes. Comparison of libraries showed that *Wolbachia* infection significantly regulated 91 miRNAs in females and 20 miRNAs in males, with an overall suppression of miRNAs in *Wolbachia*-infected libraries. A comparison of the miRNA and mRNA data predicted that the differentially expressed miRNAs negatively regulated 90 mRNAs in females and 9 mRNAs in males. An analysis of target genes showed that *Wolbachia*-responsive miRNAs regulated genes with function in sphingolipid metabolism, lysosome function, apoptosis and lipid transporting in both sexes, as well as reproduction in females.

**Conclusion:**

Comparisons of the miRNA and mRNA data can help to identify miRNAs and miRNA target genes involving in *Wolbachia*-host interactions. The molecular targets identified in this study should be useful in further functional studies.

**Electronic supplementary material:**

The online version of this article (doi:10.1186/1471-2164-15-1122) contains supplementary material, which is available to authorized users.

## Background

*Wolbachia* is a maternal transmitted endosymbiont that infect about 40% of all arthropod species as well as many filarial nematode species and has profound effects on host biology [1, 2]. *Wolbachia* manipulates host reproductive systems through a variety of strategies, including cytoplasmic incompatibility (CI), male killing, feminization, and parthenogenesis [3]. In some cases, *Wolbachia* can also provide benefits [4] and develop mutualistic relationships with their hosts [5, 6]. However, little is known about the molecular mechanisms underlying the above *Wolbachia*-mediated phenotypes in hosts.

MicroRNAs (miRNAs) are endogenous non-coding small RNAs (~22nt) that play significant roles in regulating a range of cellular processes, including development, differentiation, apoptosis, and immunity [7–10]. Originally, miRNAs were considered to control gene expression post-transcriptionally by repressing mRNA translation or degrading mRNA in the cytoplasm [11, 12]. Recently, miRNAs was observed to induce gene expression [12, 13] and even regulate pre-mRNA processing in the nucleus, which has a significant role in transcriptional gene silencing [14–16]. To date, more than 30,000 miRNAs from over 100 organisms, including two ticks and one mite, have been discovered and deposited in miRNA database (miRBase v. 21.0; ftp://mirbase.org/pub/mirbase/21) [17].

Recent studies have shown that miRNAs are important players in response to bacterial and viral infections in animals and plants [8, 15]. In the mosquito *Aedes aegypti*, the *w*MelPop-CLA strain of *Wolbachia* significantly alters the miRNA profile of *A. aegypti*, thereby manipulating a broad array of host genes to facilitate self-maintenance [13, 16, 18–20]. These observations indicate that host miRNAs may play essential role in *Wolbachia*-host interactions.

The two-spotted spider mite *Tetranychus urticae* is a cosmopolitan agricultural pest that feeds on hundreds of plant species [21]. We previously demonstrated that *Wolbachia* induces strong CI and increases host fecundity in *T. urticae* [22]. In order to explore whether and how host miRNAs are involved in *T. urticae*-*Wolbachia* interactions, we constructed four small RNA (sRNA) libraries representing female mites infected (FI) and uninfected (FU) with *Wolbachia*, and male mites infected (MI) and uninfected (MU) with *Wolbachia*. These libraries also offer the opportunity to identify protein-encoding genes that were differentially expressed in response to *Wolbachia* infection. By integrating the miRNA and mRNA data, the target genes of the differentially expressed miRNAs were identified. Furthermore, several altered miRNAs associated with reproduction were analyzed. Our results provide new insights on how *Wolbachia* affects its hosts through mediating host miRNAs.

## Results and discussion

### Identification of known and novel miRNAs in *T. urticae*

**Figure 1:**
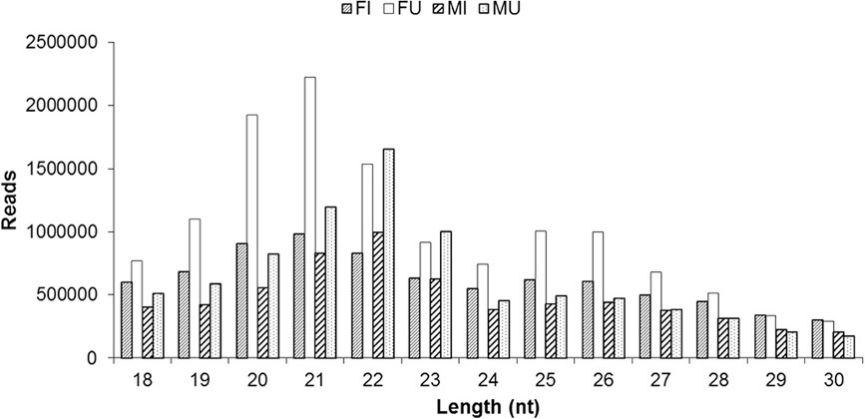
**The length distribution of small RNA (sRNA) in the four libraries.** FI, female infected; FU, female uninfected; MI, male infected; MU, male uninfected; nt, nucleotides.

**Figure 2:**
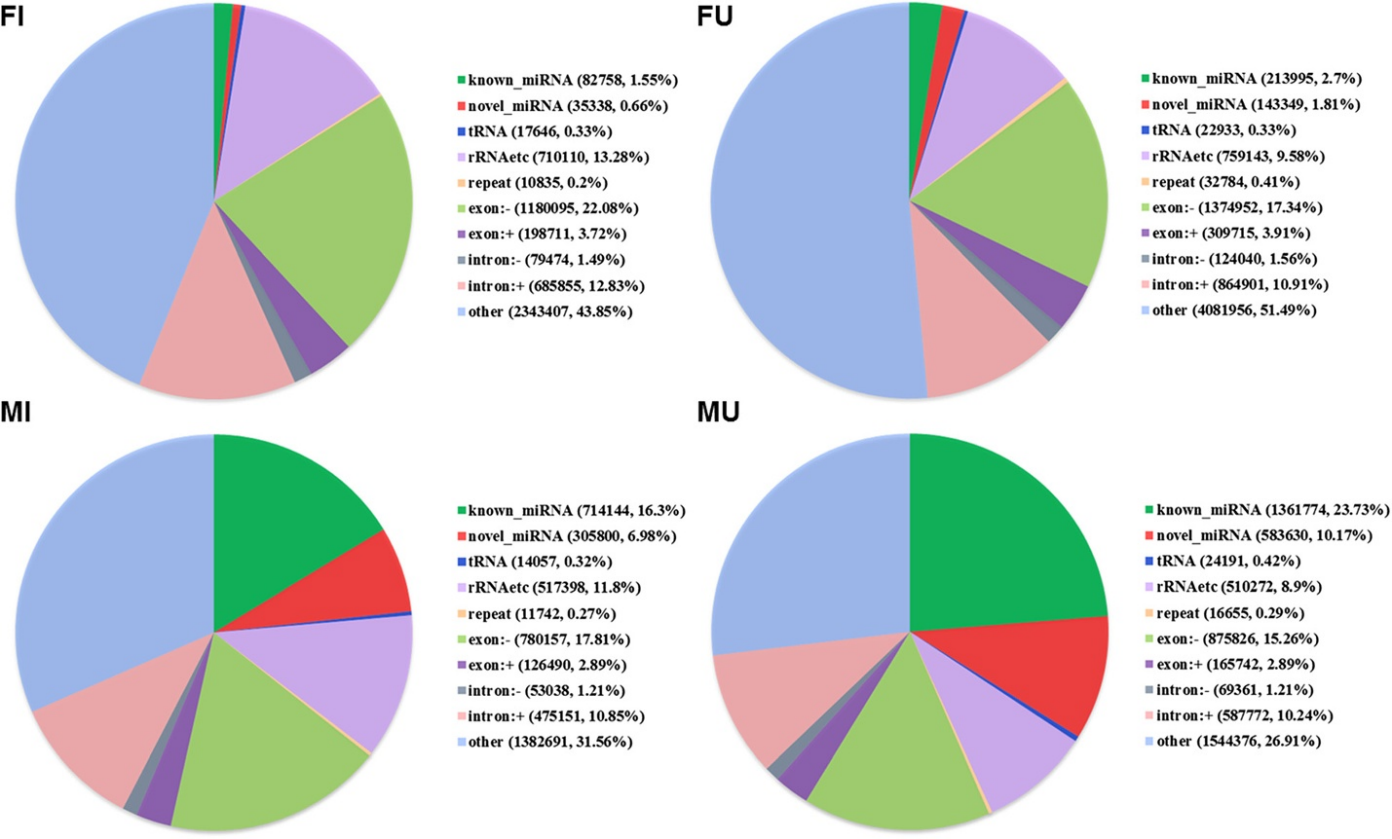
**Classification of sRNAs in the four libraries.** ‘rRNAetc’ includes rRNA, snRNA (small nuclear RNA) and snoRNA (small nucleolar RNA); others, unannotated sRNA.

Four sRNA libraries were constructed with a total of about 49 million raw reads. After sequencing process, 48 million clean reads were generated (Table 1). About 80% of the sRNAs were in the range 18-25nt and the peak sizes were 21nt for the FI and FU libraries and 22nt for the MI and MU libraries (Figure 1). The sRNAs were divided into ten classes, such as known miRNAs, novel miRNAs and tRNAs (Figure 2). The percentages of known and novel miRNAs were highest in the MU library (23.73% and 10.17%, respectively), but lowest in the FI library (1.55% and 0.66%, respectively).

**Table 1:** **Statistics of small RNA sequences of the four libraries**

Group of reads	Number of sequences				
	FI ^a^	FU ^b^	MI ^c^	MU ^d^	Total
Raw data	11,861,957	17,187,650	9,199,249	11,176,586	49,425,442
Clean reads	11,578,165	16,880,317	8,970,362	10,940,776	48,369,620
Mapped total reads	8,030,951	13,056,997	6,232,790	8,300,393	35,621,131
Mapped total sRNA	82,758	213,995	714,144	1,361,774	2,372,671
Mapped mature^e^	76	80	79	78	83
Novel miRNA	88	108	83	105	112
Novel miRNA*	31	45	29	46	67

^a^That is female infected; ^b^That is female uninfected; ^c^That is male infected; ^d^That is male uninfected. ^e^That indicates known miRNAs. *That indicates passenger strand.

A total of 83 known miRNAs and 112 novel miRNAs were identified in the four libraries (Table 1). Most of the predicted novel miRNAs had the guide strand retained and lost the passenger strand (miRNA*) (data not shown), so only the guide strand of a novel miRNA was analyzed further in this study. These novel miRNAs showed all characteristic signatures of a miRNA as described previously [23]. The predicted secondary structures of nine novel miRNAs were shown in Additional file 1: Figure S1. About 45% of novel miRNAs were 25nt and 77% of the novel miRNAs were below 100 readcount (Additional file 2: Table S2). According to miFam.dat (http://www.mirbase.org/ftp.shtml) and Rfam [24], forty conserved miRNAs and five novel miRNAs (novel_10, novel_62, novel_65, novel_67 and novel_112) were classified into 31 miRNA families (Additional file 2: Table S2).

**Figure 3:**
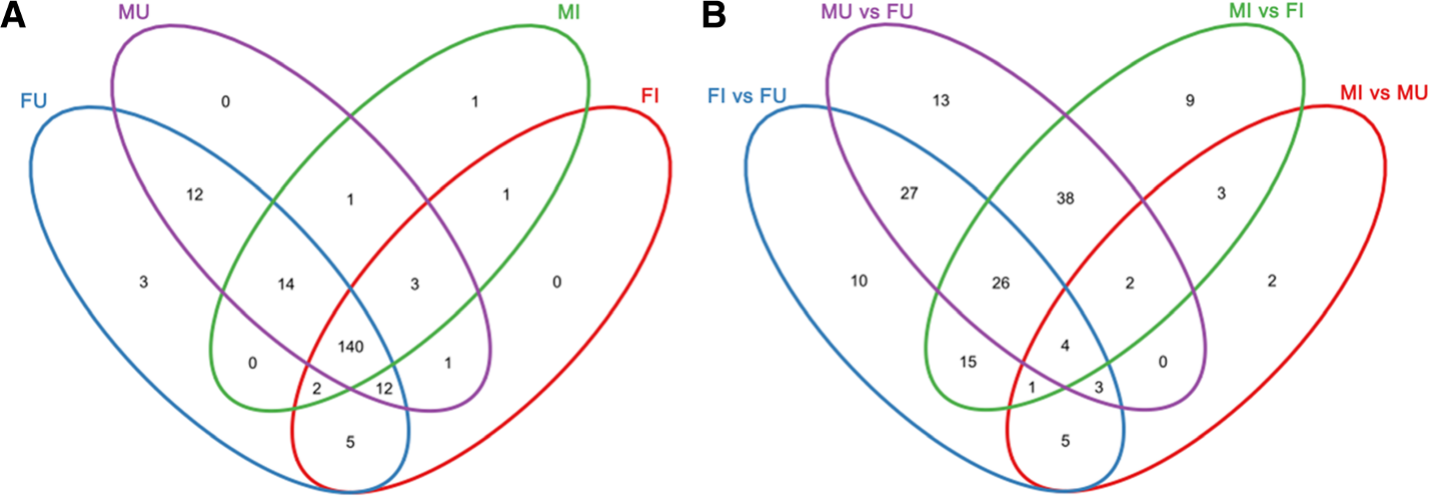
**Venn chart of miRNA expression in the four libraries. A**. Unique and commonly expressed miRNAs among four libraries (FI, FU, MI and MU). Blue oval represents FU; magenta oval represents MU; green oval represents MI; red oval represents FI. **B**. Unique and commonly differentially expressed miRNAs among four comparisons (FI vs FU, MI vs MU, FI vs MI, FU vs MU). Blue oval represents FI vs FU; magenta oval represents MU vs FU; green oval represents MI vs FI; red oval represents FI vs MU.

There were significant overlaps on identified miRNA between different groups (Figure 3A). Specifically, 193 miRNAs were identified in female libraries. Among them, 5 miRNAs were detected only in FI and 29 miRNAs were found only in FU, while 159 miRNAs were found in both libraries (Figure 3A). 189 miRNAs were identified in the male libraries, with 158 miRNAs shared by MI and MU libraries. Similar to the observation that less miRNAs present in FI than FU, the MI and MU libraries had 4 and 25 library-specific miRNAs, respectively (Figure 3A). These results indicate that the presence of *Wolbachia* may inhibit the synthesis of miRNAs in both female and male mites.

### Abundance of miRNAs

miRNAs with more than 1,000 transcripts per million (TPM) were classified as abundant while those with less than 10 TPM were classified as rare. The 20 most abundant miRNAs in each of the libraries (accounting for ~90% of total miRNA reads) are listed in Table 2. Three of them (novel_85, novel_1 and novel_2, shown in bold) were novel miRNAs. The number of rare miRNAs in FI (52) was twice the number of FU (26), while showed no significant difference between MI (91) and MU (90), indicating that many miRNAs may be down-regulated in infected females (See Additional file 2: Table S2).

**Table 2:** **Top 20 most abundant miRNAs expressed in the four libraries (readcount were shown)**

miRNA	FI	miRNA	FU	miRNA	MI	miRNA	MU
**novel_85**	**22619**	**novel_85**	**98938**	**novel_85**	**269413**	**novel_85**	**499932**
tur-miR-5735-3p	13286	tur-miR-5735-3p	57188	tur-miR-5735-3p	201373	tur-miR-5735-3p	293267
tur-miR-1-3p	9143	**novel_1**	29435	tur-miR-1-3p	108549	tur-miR-1-3p	222800
tur-miR-184-3p	9108	tur-miR-1-3p	20829	tur-miR-184-3p	92538	tur-miR-184-3p	160769
**novel_1**	**7686**	tur-miR-184-3p	19575	tur-miR-263a-5p	48966	tur-miR-263a-5p	126630
tur-miR-9-5p	6924	tur-miR-9-5p	14606	tur-miR-263b-5p	44050	tur-miR-263b-5p	118166
tur-miR-263a-5p	4236	tur-miR-7-5p	10830	tur-miR-3931-3p	33181	tur-miR-276-3p	73344
tur-miR-281-3p	3894	tur-miR-263a-5p	9885	tur-miR-7-5p	32132	tur-miR-7-5p	59615
tur-miR-7-5p	3732	tur-miR-34-5p	9589	tur-miR-276-3p	27879	**novel_1**	**59549**
tur-miR-276-3p	3448	tur-miR-281-3p	9278	**novel_1**	**23606**	tur-miR-3931-3p	42334
tur-miR-34-5p	2756	tur-miR-263b-5p	8319	tur-miR-9-5p	18715	tur-miR-9-5p	40534
tur-miR-317-3p	2654	tur-miR-276-3p	6807	tur-miR-10-5p	10482	tur-miR-71-3p	24190
tur-miR-263b-5p	2163	tur-miR-317-3p	4038	tur-miR-87-3p	7974	tur-miR-10-5p	20772
tur-miR-2-3p	1924	tur-miR-3931-3p	3559	tur-miR-34-5p	7795	tur-miR-71-5p	16812
tur-miR-133-3p	1809	tur-miR-71-3p	3418	tur-miR-71-3p	6724	tur-miR-34-5p	16357
tur-miR-190-5p	1623	**novel_2**	**3380**	tur-miR-1-5p	6543	tur-miR-190-5p	14813
tur-miR-305-5p	1590	tur-miR-12a-3p	3355	tur-miR-305-5p	6424	tur-miR-279-3p	13506
tur-miR-3931-3p	1482	tur-miR-281-5p	3156	tur-miR-281-3p	5822	tur-miR-281-3p	13479
tur-miR-71-3p	1245	tur-miR-133-3p	3136	tur-miR-133-3p	5319	tur-miR-10-3p	12549
tur-miR-12a-3p	1239	tur-miR-87-3p	3012	tur-miR-10-3p	5207	tur-miR-87-3p	12098

Novel miRNAs are shown in bold.

### Expression analysis of *Wolbachia*-responsive miRNAs

**Figure 4:**
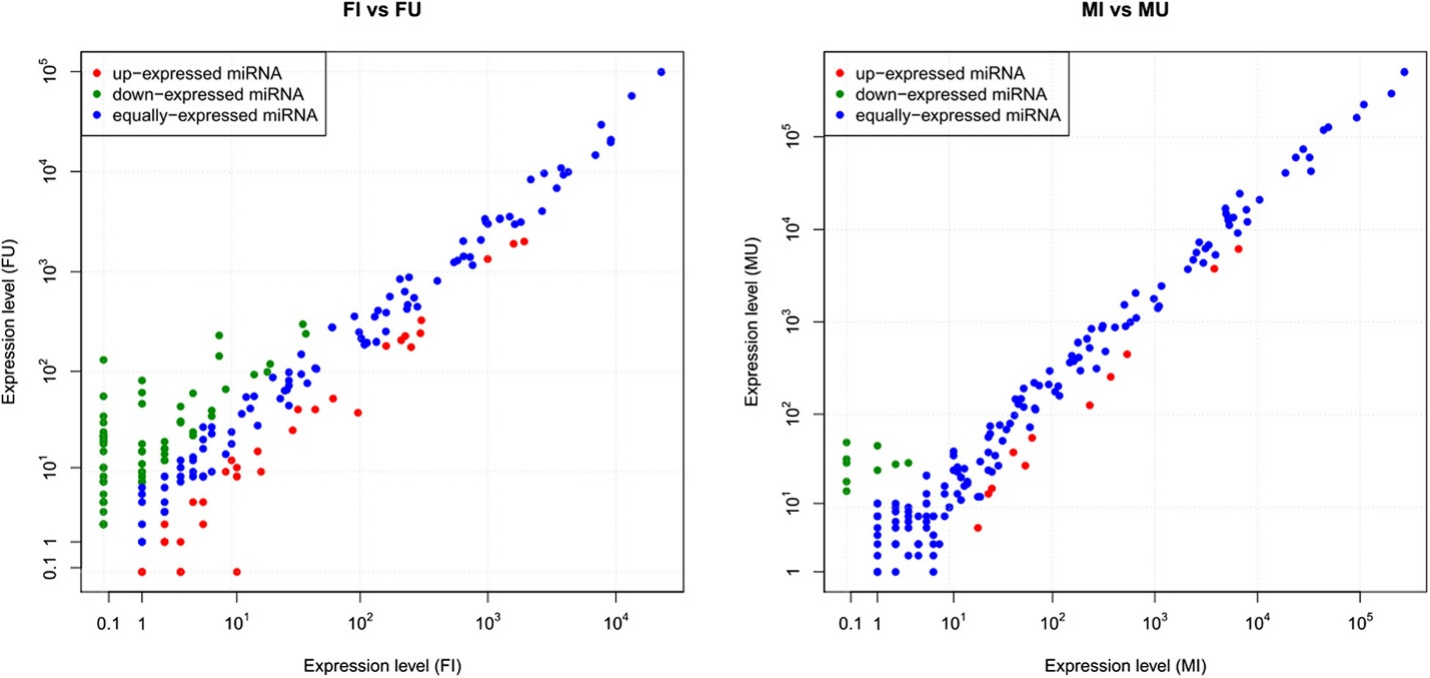
**Expression of miRNAs in FI vs FU and MI vs MU.** The x-axis and y-axis show the expression levels of miRNAs in each library. Each point represents a miRNA. Red points represent the up-regulated miRNAs. Blue points indicate equally-expressed miRNAs. Green points represent down-regulated miRNAs. FU or MU was set as control.

In females, 32 miRNAs were observed to be up-regulated and 59 down-regulated in the *Wolbachia*-infected line compared to the *Wolbachia*-uninfected line (Additional file 3: Table S3, Figure 4), while 11 miRNAs were up-regulated and 9 down-regulated in the *Wolbachia*-infected line in males (Additional file 4: Table S4, Figure 4). Among the up-regulated miRNAs, tur-miR-1-5p and tur-miR-2-3p were strongly expressed in MI and FI, respectively, while they were not among the top 20 miRNAs in MU and FU (Table 2). tur-miR-2-3p belongs to the mir-2 miRNA family, which is widespread in invertebrates, and is the largest family of miRNAs in the model species *Drosophila melanogaster* [17, 25]. Based on their predicted targets, mir-2 miRNAs have been suggested to have a role in neural development and maintenance [26]. Leaman *et al.* [27] showed that the miR-2 family regulates cell survival by translational repression of proapoptotic factors. Thus, the up-regulation of tur-miR-2-3p in *Wolbachia*-infected females may be related to changes in neural or apoptotic processes.

Thirteen differentially expressed miRNAs were common between the female and male (overlap of blue and red ovals in Figure 3B). Among them, novel_23 was up-regulated in *Wolbachia*-infected males but down-regulated in *Wolbachia*-infected females (Table 3). Four other miRNAs were up-regulated in *Wolbachia*-infected lines, and eight miRNAs were down-regulated in *Wolbachia*-infected lines (Table 3). Interestingly, novel_126, novel_23 and novel_32 were detected only in *Wolbachia*-uninfected females (See Additional file 3: Table S3). Similarly, novel_105, novel_109, novel_126, novel_16 and novel_32 were found only in *Wolbachia*-uninfected males (Additional file 4: Table S4).

**Table 3:** **Predicted target genes of the 13 common** ***Wolbachia*** **-responsive miRNAs in the female and male comparisons**

miRNA name	log _2_(FI/FU) ^a^	log _2_(MI/MU) ^a^	Target Gene id	log _2_(FI/FU) ^b^	log _2_(MI/MU) ^b^	Gene description	Pathway ^c^
novel_126	-9.6029	-4.9958	tetur13g00510	2.7968	--	Histone H2B (Fragments)	
novel_32	-7.6988	-4.1657	tetur19g00840	2.7184	2.688	-//-	
novel_23	-4.8914	2.8724	tetur19g00830	2.9756	--	Tetratricopeptide repeat protein 19, mitochondrial	
			tetur10g01260	--	-3.5643	Retrovirus-related Pol polyprotein from transposon 412	
novel_105	-4.8984	-4.8537	tetur01g12840	1.9333	--	Adult-specific rigid cuticular protein 15.7	
			tetur11g00600	1.1574	--	Cuticle protein 10.9	
novel_36	-4.4893	-3.5605	tetur11g00940	6.2399	--	Meiosis arrest female protein 1	
novel_16	-4.1131	-5.6105	tetur09g06680	4.1748	2.2697	Glucosylceramidase	YES
			tetur26g00300	4.047	4.5209	Protein lifeguard 1	
novel_89	-3.5904	-4.4674	tetur19g00830	2.9756	4.0633	Tetratricopeptide repeat protein 19, mitochondrial	
novel_109	-1.9278	-3.8031	tetur04g01490	2.5622	--	-//-	
			tetur09g04610	--	1.0918	Multidrug resistance-associated protein 1	
novel_43	-1.1435	-2.2486	tetur03g00110	2.9146	--	Protein lethal (2) essential for life	YES
			tetur04g01580	1.9481	--	-//-	
			tetur28g01280	2.3157	--	Beta-1,3-galactosyltransferase sqv-2	YES
			tetur87g00010	2.5029	--	Nuclear hormone receptor HR96	
			tetur09g04610	--	1.0918	Multidrug resistance-associated protein 1	
tur-miR-12b-5p	1.2635	1.1341	tetur06g06620	-2.1594	--	-//-	
			tetur26g00570	-8.9015	--	Low-density lipoprotein receptor-related protein 8	
novel_33	1.4415	2.8724	--				
tur-miR-317-5p	1.5433	1.2203	--				
tur-miR-745-3p	1.6556	2.0244	--				

^a^The columns two and three show the log2 (fold-change) of miRNAs; ^b^The columns five and six show the log2 (fold-change) of mRNAs. ^c^Three target genes have been enriched in the KEGG pathways.

We also identified 74 female-specific differentially expressed miRNAs, including 18 substantially (decreased by more than 16-fold) down-regulated miRNAs and 5 substantially up-regulated miRNAs (increased by more than 16-fold) (Additional file 3: Table S3). In *Wolbachia*-infected females, the most down-regulated miRNA was novel_126, which had a log2 (fold-change) value of 9.603. In contrast, novel_59 was most up-regulated with a log2 (fold-change) value of 7.332 (Additional file 3: Table S3). Furthermore, several miRNAs like novel_59, novel_117, novel_45, novel_120 and tur-miR-5729a-3p were found only in infected females (Additional file 3: Table S3).

### miRNA expression patterns

**Figure 5:**
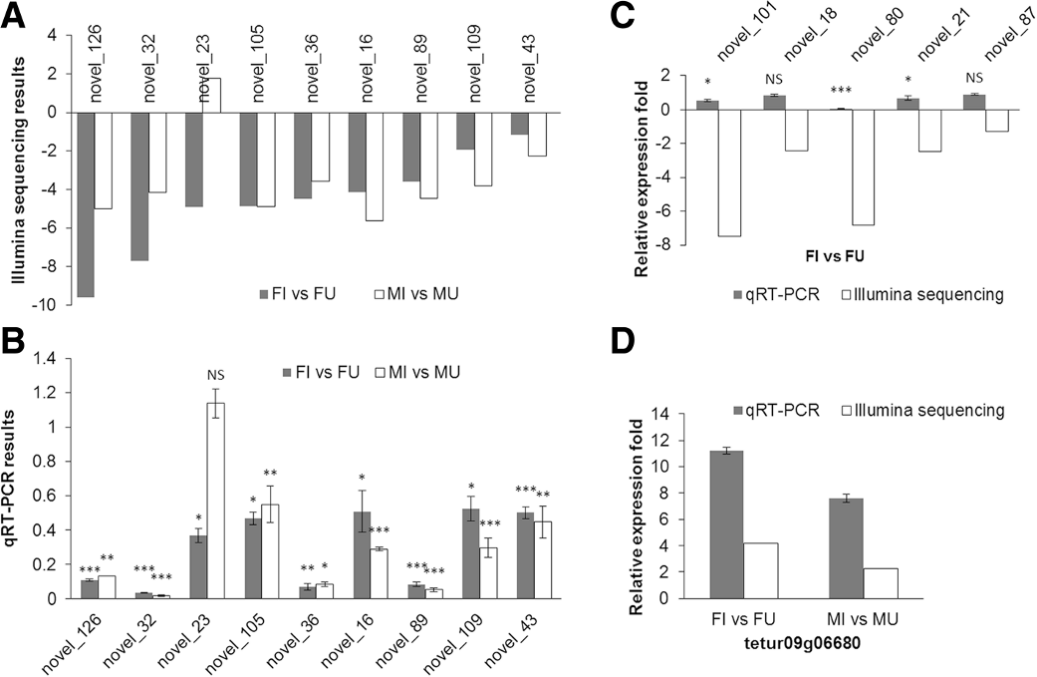
**Expression patterns of differentially expressed miRNAs and one target gene (tetur0906680) in the female and male comparisons. A**. and **B**. Nine common differentially expressed miRNAs in the comparisons FI vs FU (control) and MI vs MU (control) identified by using Illumina small RNA deep sequencing (log2 fold-change) and validated by qRT-PCR. **C**. Validation of five female-specific differentially expressed miRNAs by qRT-PCR. **B** and **C**. Error bars represent standard errors of averages from three biological replicates (NS: not significant; *P < 0.05; **P < 0.01; ***P < 0.001; Fisher's LSD test). **D**. qRT-PCR and Illumina small RNA deep sequencing both showed that candidate target gene tetur09g06680 of novel_16 was up-regulated in *Wolbachia*-infected mites. All the experiments had three biological replicates.

To further validate the expression patterns of miRNAs identified in this work, we measured the expression levels of nine differential miRNAs (novel_126, novel_32, novel_105, novel_36, novel_16, novel_89, novel_109, novel_43 and one novel_23) shared by the female and male and five female-specific differential miRNAs (novel_101, novel_18, novel_80, novel_21 and novel_87) using quantitative real time polymerase chain reaction (qRT-PCR). Except for novel_23, all other 8 miRNAs were significantly (*P* < 0.05) down-regulated in *Wolbachia*-infected female and male lines, which was in agreement with our sequencing data (Figure 5A,B). novel_126, novel_32, novel_36 and novel_89 were decreased by more than five times (*P* < 0.001). Three of the five female-specific differential miRNAs were significantly down-regulated in infected females, which was consistent with our sequencing data (Figure 5C). As a whole, these results supported that the Illumina sequencing data accurately reflected the abundance of the miRNAs.

### Target gene prediction of differentially expressed miRNAs

To further determine the biological functions of the differentially expressed miRNAs, we integrated the miRNA and the mRNA transcriptome data to predict the candidate target genes. At first, miRanda was used to predict the target genes of the 195 miRNAs, which resulted in the identification of 41,914 miRNA-target pairs. Then, the predicted target genes of differentially expressed miRNAs were aligned with differentially expressed mRNAs. This approach revealed possible interactions between 115 mRNAs and 79 miRNAs in the female comparison (Additional file 5: Table S5), and between 92 mRNAs and 15 miRNAs in the male comparison (Additional file 6: Table S6). In addition, 65 differential miRNAs found in the female comparison were predicted to negatively regulate 90 candidate target genes (Additional file 3: Table S3). While in the male comparison, 9 candidate target genes were suggested to be negatively regulated by the 11 differentially expressed miRNAs (Additional file 4: Table S4).

**Figure 6:**
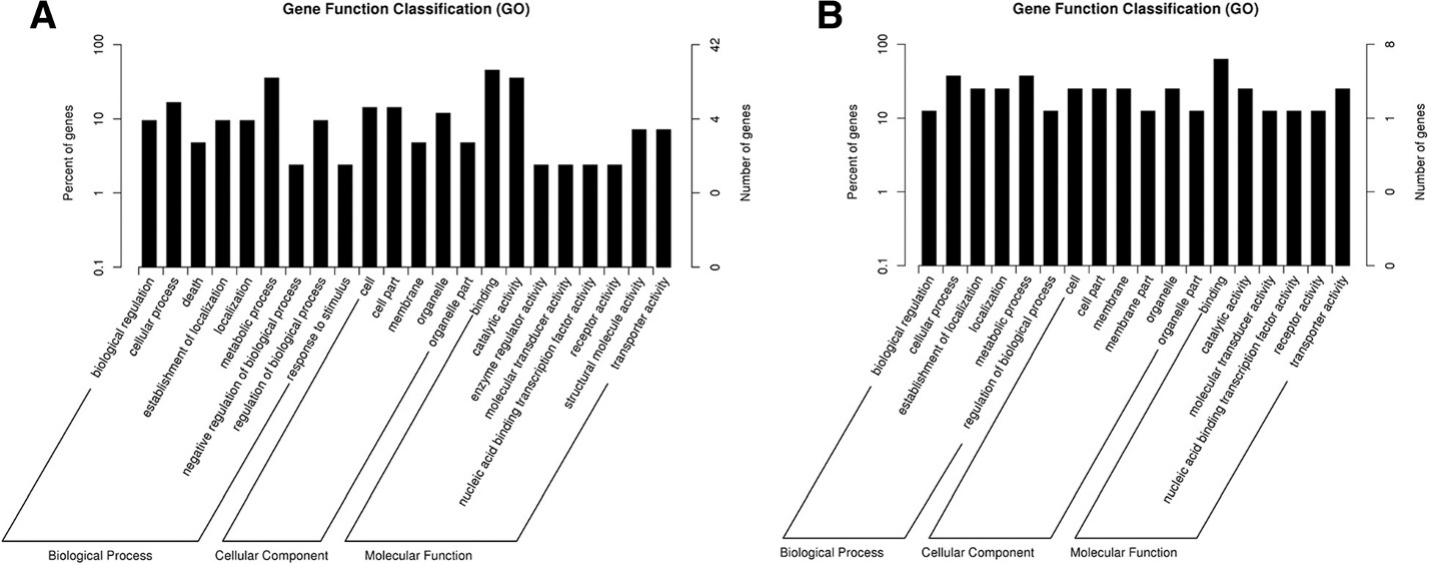
**GO analysis results of the target genes of the two comparisons. A**. FI vs FU (control); **B**. MI vs MU (control). The x-axis is the GO category and the y-axis is the percent and number of genes.

The biological functions of the predicted target genes were then analyzed using the gene ontology (GO) annotations of the *T. urticae* genes. The GO annotation enrichment results showed that genes related to binding, catalytic activity, and metabolic and cellular processes were most enriched in the two comparisons (Figure 6A and B). In both female and male mites, GOs corresponding to the up-regulated genes included establishment of localization, membrane and transporter activity (data not shown). However, several GOs, such as death, enzyme regulator activity and structural molecular activity, were exclusively enriched in the female mites (Figure 6A and B).

**Figure 7:**
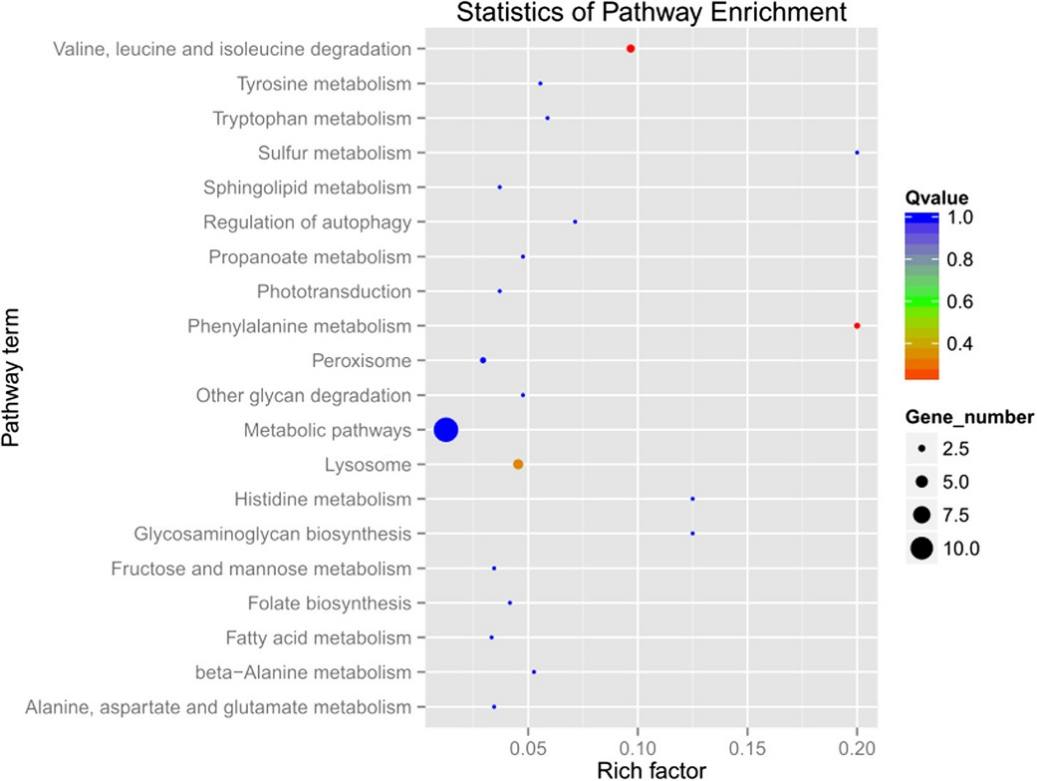
**The 20 most enriched KEGG pathways based on target genes of differentially expressed miRNAs in the female comparison.** The x-axis shows the rich factor. The y-axis shows the pathway names. The size of each point represents the number of genes enriched in a particular pathway. The bigger the value of rich factor and the smaller the value of Q-value indicate the degree of enrichment is more significant.

Pathway analysis resulted in observation of 28 and 5 different pathways corresponded to the differentially expressed genes in the female and male comparison, respectively. Notably, the five KEGG pathways (lysosome, sulfur metabolism, glycan degradation, sphingolipid metabolism and metabolic pathways) and their related three candidate genes (tetur08g05010, tetur09g06680 and tetur03g03470) of the male comparison were also highlighted in the female comparison. The 20 most enriched KEGG pathways of females included the degradation of valine, leucine and isoleucine, phenylalanine metabolism, lysosome function and so on (Figure 7). The target gene tetur09g06680 was the only enriched gene in both female’s and male’s KEGG pathways which may be negatively regulated by a differential miRNA (novel_16). Sequencing and qRT-PCR results showed that tetur09g06680 was up-regulated in both infected female and male mites (Figure 5D). The up-regulation of tetur09g06680 indicated that novel_16 potentially represses the expression of this gene.

### *Wolbachia*-responsive miRNAs are related to regulation of apoptosis in mites of both sexes

The target genes that may be negatively regulated by the 13 common differentially expressed miRNAs in the female and male comparisons were presented in Table 3. In general, the target genes of some miRNAs revealed multiply. Despite the similarities in miRNAs regulation, we found that only novel_16 and novel_89 targeted the same genes in both comparisons (Table 3). The miRNA novel_16 targets two genes potentially associated with apoptosis: tetur09g06680 encoding glucosylceramidase and tetur26g00300 encoding protein lifeguard 1, which were up-regulated in *Wolbachia* infected mites with more than 4-fold (Table 3). The gene tetur09g06680 is involved in several KEGG pathways, including lysosome function, sphingolipid metabolism and glycan degradation (data not shown). Deficiency of GCase causes several diseases in mice, including severe neurodegeneration and apoptosis in the brain [28, 29]. Therefore, the observed up-regulation of tetur0906680 in both infected lines may indicate its role in inhibiting apoptosis. Moreover, some other miRNAs, such as novel_100 (targets tetur03g00110), novel_84 (targets tetur26g00300), novel_92 (targets tetur03g00110), novel_50 (targets tetur26g00300), may also regulate apoptosis (Additional file 3: Table S3). The expression level of tetur03g00110, encoding Protein lethal (2) essential for life, was elevated in *Wolbachia*-infected females. Previous studies have proved that *Wolbachia* can inhibit apoptosis in several ways. For example, *Wolbachia* surface protein (Wsp) inhibits apoptosis of Polymorphonuclear (PMN) cells *in vitro* [30]. In the parasitic wasp *Asobara tabida*, *Wolbachia* helps its host to avoid becoming reproductively sterile by repressing massive apoptosis of nurse cells in the ovary [31]. Similarly, *Wolbachia* infection leads to a decrease in programmed cell death in the germarium of *D. mauritiana* [32]. However, a virulent *Wolbachia* strain *w*MelPop increase the frequency of apoptosis in the female *Drosophila* [33]. Regardless of the strategy that *Wolbachia* uses to interfere with host, the apoptosis inhibition will in turn benefit to maintenance of *Wolbachia* itself. The down-regulation of the anti-apoptotic miRNAs (novel_16, novel_100, novel_84, novel_92 and novel_50) reinforces the importance of investigating the apoptotic signaling cascade upon *Wolbachia* infection (Additional file 3: Table S3 and Additional file 4: Table S4).

### *Wolbachia*-responsive miRNAs may regulate reproduction in female mites

Since *Wolbachia* causes strong CI in this spider mite population, an important aim of our study was to clarify the underlying mechanism of *Wolbachia*-induced CI. Some remarkable features observed in CI embryos include delayed paternal nuclear envelope breakdown and activation of Cdk1 [34], a failure of the maternal histones H3.3 to deposit in the paternal genome, and slowed replication of sperm DNA [35]. In this study, we found several target genes that may be related to females’ reproduction. For instance, target gene tetur13g00510 (targeted by novel_126) codes for Histone H2B was induced by 6.9-fold in *Wolbachia*-infected females (Table 3). Histone play important roles in transcription regulation, DNA repair, DNA replication and chromosomal stability [36]. Target gene tetur11g00940 of novel_36 was exclusively expressed in *Wolbachia*-uninfected females (Additional file 5: Table S5), which codes for meiosis arrest female protein 1 and involved in oogenesis (Table 3). Target gene tetur11g00600 and tetur01g12840 (targeted by novel_105) code for cuticle protein, which may be related to egg formation (Table 3). In addition, target gene tetur04g05770 (potentially regulated by novel_80) codes for sorbitol dehydrogenase, an enzyme which possibly play a role in sperm motility by providing a source of energy for sperm [37] (Additional file 3: Table S3). Taken together, it supports the hypothesis that *Wolbachia* can manipulate female mites’ reproduction via host miRNAs and further studies are needed to test this hypothesis.

### Other function genes mediated by *Wolbachia*-responsive miRNAs

Among the seven male-specific differential miRNAs, only two had target genes with description (Additional file 4: Table S4). The target gene exclusively found in the male comparison was tetur09g04610 (targeted by novel_109), which codes for multidrug resistance-associated protein 1 (Table 3). The target gene of tur-miR-9-3p (tetur09g04720) codes for apolipoprotein D, which is related to lipid transporting. We also found target genes related to drug resistance (gene tetur10g01260 potentially regulated by novel_80) and apolipophorins biogenesis (gene tetur20g01230 potentially regulated by novel_100) were up-regulated in the infected females (Additional file 3: Table S3), which indicated that these two biological processes may be regulated by different miRNA-target pairs in the female and male mites. Apolipophorins may be up-regulated in female mites because they are essential for reproduction, but they may also be up-regulated by *Wolbachia* because *Wolbachia* needs them to produce lipopolysaccharide, the major component of its outer membrane [38]. Another observation worth to note here was that *Wolbachia* also enhances the expression of the genes that codes for riboflavin transporter in female mites (Additional file 3: Table S3). Riboflavin that *Wolbachia* provided to nematodes was found to be crucial for worm health and fertility (6), and in cultured mosquito cells, depletion of host cell riboflavin decreased *Wolbachia* abundance [39]. Taken together, these results raise the possibility that *Wolbachia* and *T. urticae* have a symbiotic relationship.

### Sex-specific interaction mechanisms

The divergences of the differentially expressed miRNAs and target genes found in the female and male comparisons suggest that *Wolbachia* used different strategies to regulate miRNA and mRNA in females and males. We previously showed that *Wolbachia* mainly localized in the ovary of female mites and its density increased during the reproduction process [22]. In contrast, the density of *Wolbachia* in male mites decreased with age [22]. These phenomena indicated that *Wolbachia* replicate massively in female reproductive tissues to ensure its successful vertical transmission. In the present study, *Wolbachia* infection led to the down-regulation of 65% of the differential miRNAs and up-regulation of 69% of the predicted target genes in the female mites (Additional file 3: Table S3). GO analysis showed that the up-regulate genes were related to establishment of localization, membrane, structural molecular activity and transporter activity (data not shown), which also suggested that *Wolbachia* replication is increasing in young females. Besides *Wolbachia* replication, the reproductive tissues of female mites probably experience high metabolic activity during egg production. That could be two reasons why there are more specific- and up- regulated mRNAs in females. Some studies suggested that *Wolbachia* interacts with female hosts in a broader manner than it does with male hosts. In some cases, *Wolbachia* acts as an obligate mutualism, i.e., it is required for normal reproduction of the host [5, 40]. In other cases, *Wolbachia* was found to benefit to its female host by increasing fecundity [4, 22]. Sex-specific regulations of miRNAs were also found in flour beetles, in which 245 (54%) of the miRNAs exhibited gender-specific expression patterns upon exposure to environmental stress [41]. This discrepancy in gender- specific miRNA expression is consistent with Bateman’s principle, which states that males gain fitness by increasing their mating success whereas females gain fitness through increasing longevity because their reproductive effort is higher [42]. If the female and male mites gain fitness through different ways following *Wolbachia* infection, the expression of miRNAs and mRNAs may be diversely influenced.

## Conclusion

Four libraries of young female and male mites with and without *Wolbachia* infection, were constructed, amplified and sequenced. As a result, we detected 91 and 20 miRNAs exhibiting differential expression in response to *Wolbachia* infection in female and male mites, respectively. There was an overall decrease of miRNAs in *Wolbachia*-infected lines. Integrating the miRNA and mRNA data uncovered many target genes, and enabled us to develop new hypotheses for *Wolbachia*-regulated reproduction and apoptosis mechanisms. The discovery of these *Wolbachia*-responsive miRNAs and their target genes provides a basis for understanding *Wolbachia*-host interaction in a CI phenotype host. In addition, we described a complex interaction network of miRNAs and target mRNAs that will encourage future studies to examine the contribution of the specific newly identified miRNAs on the regulation of biological processes in response to *Wolbachia* infection in more detail.

Eventually, this study reports the discovery of sexually differentially expressed miRNAs and miRNA- target gene pairs. *Wolbachia* appears to affect a large number of cellular processes in female mites. Those findings lay the foundation for future studies to identify miRNAs or genes responsible for not only the *Wolbachia* maintenance, but also the sexually host-endosymbiont interaction mechanism.

## Methods

### Two-spotted spider mite

Mites used in this study were originally collected from Hohhot, Inner Mongolia, northeast China in July 2010 and reared on leaves of the common bean (*Phaseolus vulgaris* L.) placed on a water-saturated sponge mat in Petri dishes (dia. 9) at 25 ± 1°C, 60% r. h. and under L16-D8 conditions. 100% infected and 100% uninfected *Wolbachia* lines with identical genetic backgrounds were prepared according to a method described by Xie *et al.* [43]. Through PCR assays, neither line was found to be infected with *Cardinium* (CLO-f1: 5’-GGAACCTTACCTGGGCTAGAATGTATT-3’, CLO-r1: 5’-GCCACTGTCTTCAAGCTCTACCAAC-3’) or *Rickettsia* (R1: 5’-GCTCTTGCAACTTCTATGTT-3’, R2: 5’-CATTGTTCGTCAGGTTGGCG-3’) [44], which can manipulate host reproduction. Within the two lines, 1–3 day old adult females (Turt_FI and Turt_FU, ‘I’ indicates infection and ‘U’ indicates uninfection) and 1 day-old adult virgin males (Turt_MI and Turt_MU) were respectively collected. The samples were stored in liquid nitrogen until required for RNA isolation.

### Small RNA library construction for Illumina sequencing

Total RNA was extracted by using Trizol reagent (Invitrogen catalog no. 15596–026) according to the manufacturer’s protocol with small modifications. Total RNA (>3 μg) with good quality was used to construct a sRNA library for each sample by using a TruSeq small RNA Sample Pre Kit (Illumina). Briefly, total RNA was ligated with 5’ and 3’ adaptors followed by reverse transcription using RT primers. After PCR amplification of the cDNAs, amplified PCR products within 130–160 bp were purified on an 8% polyacrylamide gel (100 V, 80 min). The library quality was assessed on the Agilent Bioanalyzer 2100 system using DNA High Sensitivity Chips and then sequenced on a HiSeq2000 sequencer (Illumina).

### Bioinformatics analysis

After Illumina sequencing, raw data were processed through Novogene Company’s Perl and Python scripts. In this step, clean data were obtained by removing reads containing more than three N (undetermined bases), with 5’ adapter contaminants, without 3’ adapter or the insert tag, containing poly A or T or G or C and low quality reads from the raw data. Then, sRNAs with lengths of 18–30 nt were selected for further analyses. In the alignment and annotation procedure, we used the following priority rule: known miRNA > rRNA > tRNA > snRNA > snoRNA > repeat > gene > novel miRNA so that every unique sRNA mapped to only one annotation. Briefly, the first step was mapping the sRNA tags to the *T. urticae* genome sequence (http://bioinformatics.psb.ugent.be/orcae/overview/Tetur; released in Sep, 2009) by Bowtie [45] without mismatch to analyze their expression and distribution on the reference sequence. Next, the mappable sRNA tags were aligned to the miRNA precursor of *T. urticae* in the miRNA database (miRBase v. 20.0; released in June, 2013) to obtain the known miRNA count. Then, tags originating from rRNAs, tRNAs, snRNAs, and snoRNAs were removed by mapping the remained sRNA tags to the ncRNA annotation database of *T. urticae* (https://bioinformatics.psb.ugent.be/gdb/tetranychus/small_RNAs/). Tags originating from repeat sequences were filtered by using a repeat sequences database (http://www.repeatmasker.org/cgi-bin/WEBRepeatMasker/), and tags originating from protein-coding genes were discarded by mapping to the exon and intron of mRNAs of *T. urticae*. Finally, novel miRNAs were predicted by exploring the secondary structure, the Dicer cleavage site and the minimum free energy of the former unannotated sRNA tags which could be mapped to the reference sequence by integrating two available software miREvo [46] and mirdeep2 [47].

### Comparison of miRNA libraries

The readcounts of miRNAs were transformed into TPM (transcript per million) through the following criteria: Normalization formula: Normalized expression = (mapped readcount/Total reads)*1000000 [48] and then analyzed the abundance of miRNAs. Since we didn’t set up biological replicates for each sample, when analyzing differentially expressed miRNAs between libraries, we first normalized the readcount data by using TMM (trimmed mean of M values) [49], then used the DEGseq R package to analyze the differences [50]. The P-value was adjusted using the q value [51]. q Value <0.01 and |log2 (fold-change)| > 1 was set as the threshold for significant differential expression by default. We compared the expression level of miRNAs between FI and FU (control), MI and MU (control), MI and FI (control), MU and FU (control).

### Target prediction

The 3’ UTR annotation information originated from the genome database of *T. urticae* (https://bioinformatics.psb.ugent.be/gdb/tetranychus/) was used by miRanda [52] to predict target genes of miRNAs. As we used the same sample to get the RNA transcriptome data (Transcriptome raw data are available in the ArrayExpress database at http://www.ebi.ac.uk/arrayexpress under accession number E-MTAB-2491), we integrated the target genes of differentially expressed miRNAs with the differentially expressed genes found by comparing the four transcriptomes to obtain more precise miRNA-mRNA correlation information. Since most miRNAs were found to repress mRNA translation or induce mRNA degradation, the datasets in our study were analyzed through intersecting elements of target genes of 1) differentially expressed miRNAs and differentially expressed mRNAs, 2) differentially down-expressed miRNAs and differentially up-expressed mRNAs and 3) differentially up-expressed miRNAs and differentially down-expressed mRNAs. The datasets obtained above were used for GO enrichment analysis [53] and KEGG enrichment analysis [54]. KOBAS [55] software was used to test the statistical enrichment of the target gene candidates in the KEGG pathways.

### miRNA expression patterns

miRNA expression patterns were established in 1-day-old virgin *Wolbachia*-infected and *Wolbachia*-free females and males. The experiment was run on an ABI 7500 thermal cycler (Applied Biosystems) with three technical replicates and three biological replicates. About 500 females and 1000 males were prepared for each biological replicate. For qRT-PCR, RNA was extracted with a miRNeasy extraction kit (Qiagen catalog no. 217004) together with RNase-Free DNase Set (Qiagen catalog no. 79254). RNA was extracted according to the manufacturer’s protocol except that after grinding with a homogenizer, the homogenate was centrifuged at 12,000 × g to remove the precipitate. RNA quality was estimated with a Nanodrop spectrophotometer and by agarose gel electrophoresis. The expression of miRNA was checked by using SYBR PrimeScript miRNA RT-PCR Kit (Takara catalog no. RR716). Total RNA (1.5 μg) was reverse transcribed and 30 ng cDNA product was put into a 20 μL quantification system. The primers used in this study are shown in Additional file 7: Table S1. The reactions were incubated at 95°C for 30 s, followed by 40 cycles of 95°C for 5 s, 60°C for 34 s. A dissociation curve was obtained to ensure that only one product was amplified after the amplification phase. The 2^-ΔΔCt^ method for relative quantification of gene expression was used to determine the level of miRNA expression and U6 snoRNA was used for normalizing the data.

### Candidate target gene expression pattern

The same RNA used for miRNA expression analysis was used for target gene expression analysis. Total RNA (1.5 μg) was reverse transcribed with SuperScript III Reverse Transcriptase (Invitrogen catalog no.18080-044) according to the instructions. SYBR Premix Ex Taq™ II (Takara catalog no. RR820A) was used to quantify the target genes by putting 30 ng cDNA products into 20 μL quantification system. The qPCR cycling parameters included 95°C for 30 s, and 40 cycles of 95°C for 5 s, and 60°C for 34 s. At the end of the PCR reaction, a melting curve was generated. β-Tubulin was used as an endogenous reference gene. The experiment was conducted three times.

### Availability of supporting data

Small RNA sequencing raw data are available in the ArrayExpress database (http://www.ebi.ac.uk/arrayexpress) under accession number E-MTAB-2606.

## Electronic supplementary material

Additional file 1: Figure S1: Predicted secondary structures of nine novel miRNAs. (TIFF 5 MB)

Additional file 2: Table S2: List of all the miRNAs identified in the four libraries. (XLS 72 KB)

Additional file 3: Table S3: Predicted target genes which may be negatively-regulated by differential miRNAs in the female comparison. (XLS 60 KB)

Additional file 4: Table S4: Predicted target genes which may be negatively-regulated by differential miRNAs in the male comparison. (XLS 29 KB)

Additional file 5: Table S5: Analysis of mRNA-miRNA pairs in the comparison FI vs FU. (XLS 90 KB)

Additional file 6: Table S6: Analysis of mRNA-miRNA pairs in the comparison MI vs MU. (XLS 68 KB)

Additional file 7: Table S1: Primers used in this study. (XLS 26 KB)
