# Supplementary figures and images for: Identification of Wolbachia-responsive microRNAs in the two-spotted spider mite, Tetranychus urticae
Source: BMC Genomics. 2014 Dec 16;15(1):1122. doi: 10.1186/1471-2164-15-1122 (PMC4378230; doi:10.1186/1471-2164-15-1122)

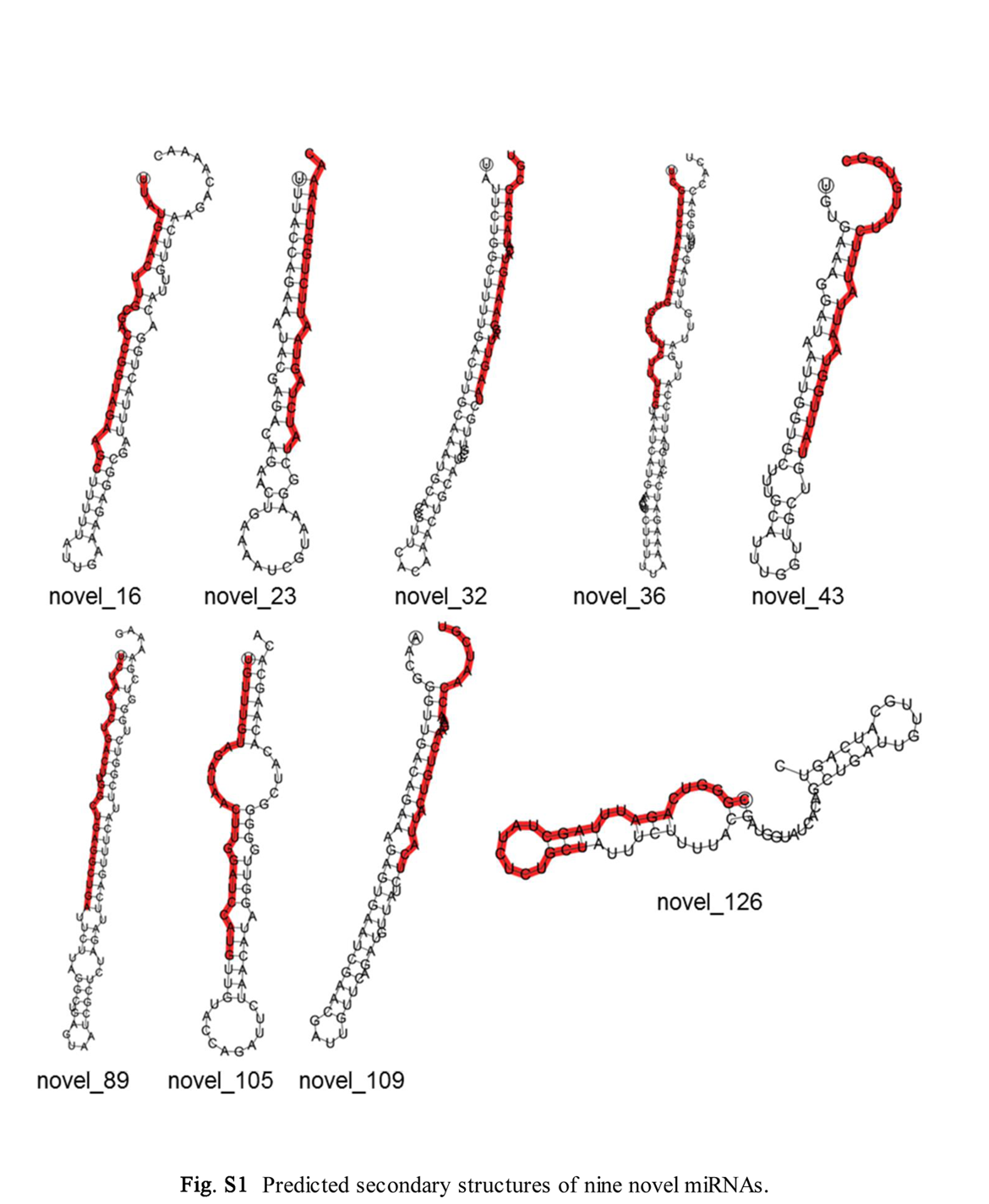

Supplement: Supplementary file 1 — Additional file 1: Figure S1: Predicted secondary structures of nine novel miRNAs. (TIFF 5 MB) [file 12864_2014_6825_MOESM1_ESM.tiff]
